# Anesthetic management in a child with Meier-Gorlin syndrome: a case report

**DOI:** 10.1186/s12871-025-03449-5

**Published:** 2025-10-27

**Authors:** Christine Gaik, Kalvis Kreiss, Astrid Morin, Katharina Toussaint, Hinnerk Wulf, Nicolas Schmitt

**Affiliations:** 1 Philipps-Universität Marburg, Marburg, Germany; 2 Department of Anesthesiology and Intensive Care Medicine, University Hospital Giessen and Marburg, Campus Marburg, Baldingerstraße, Marburg, 35033 Germany

**Keywords:** Pediatric anesthesia, Case report, Meier-Gorlin syndrome, Difficult airway

## Abstract

Meier-Gorlin syndrome (MGS) is a rare autosomal recessive disorder characterized by the clinical triad of microtia, patellar hypo- or aplasia, and proportionate short stature. Due to its phenotypic variability and frequent craniofacial anomalies, anesthetic management may be challenging. We report the perioperative management of a 5-year-old boy with genetically confirmed MGS undergoing adenoidectomy, otomicroscopy, and bilateral tympanostomy tube insertion. Preoperative airway evaluation was unremarkable, with no classical predictors of a difficult airway. However, videolaryngoscopy during induction revealed an anterior and deeply positioned glottis with soft, collapsible surrounding structures. Initial intubation with a flexible stylet was unsuccessful; a second attempt using a stiffer stylet was successful under direct visualization. The remaining perioperative course, including extubation and postoperative monitoring, was uneventful. A multidisciplinary planning and individualized anesthetic management enabled safe ambulatory care. To our knowledge, this is the first published case describing anesthetic management in a patient with MGS.

## Introduction

Meier-Gorlin syndrome (MGS) is a rare autosomal recessive disorder classified as a form of primordial dwarfism. It is clinically defined by the triad of microtia, patellar hypo- or aplasia, and proportionate short stature – although not all patients exhibit all three core features [1]. Additional manifestations may include feeding and respiratory difficulties, mammary hypoplasia, and urogenital anomalies [1].

To date, variants in 13 genes involved in DNA replication have been associated with MGS [2]. MGS was first described by Meier in 1959, further characterized by Gorlin et al. in 1975, and formally named by Boles et al. in 1994 [3–5].

The estimated prevalence is fewer than 1–9 per 1,000,000 individuals, with fewer than 100 cases reported in the literature [1, 2]. However, underdiagnosis is likely due to the wide phenotypic variability and lack of clinical awareness. Management is symptomatic and requires a multidisciplinary approach tailored to the individual’s clinical presentation, with particular emphasis on early detection and prevention of complications [1]. Given the phenotypic diversity and potential airway and musculoskeletal challenges, anesthetic management requires thorough preoperative evaluation and individualized planning. Published anesthetic data are extremely limited, highlighting the lack of evidence-based recommendations for perioperative care. Table 1 summarizes reported clinical features of MGS that may be relevant for anesthetic management (Table 1).

**Table 1:** Reported clinical features of Meier-Gorlin syndrome (MGS) with potential relevance for anesthetic management. Since the clinical presentation of MGS can vary considerably and many patients exhibit very few of these features, while some characteristics have only been described in isolated case reports, each patient should be assessed individually for syndrome-specific characteristics (according to references [1, 2], 6– [22])

System/feature	Potential anesthetic relevance
Craniofacial/oral	
Craniofacial dysmorphism (microtia, mandibular/maxillary hypoplasia, retro-/micrognathia, microstomia, ankyloglossia)	Potential difficult airway (mask ventilation, laryngoscopy, intubation, or supraglottic airway placement)
Reduced mouth opening, temporomandibular joint anomalies	Limited airway access; potential difficult laryngoscopy
Impaired dental status	Risk of dental injury during laryngoscopy or intubation; careful airway instrumentation required
Cleft (hard and soft) palate, high arched palate, bifid uvula or similar malformations	Potential difficult airway (mask leak, difficult laryngoscopy or intubation); risk of regurgitation and aspiration
Airway/pulmonary	
Airway anomalies (laryngo-, tracheo-, bronchomalacia; tracheoesophogeal fistula)	Risk of dynamic airway collapse; potentially increased susceptibility to perioperative respiratory complications
Pulmonary manifestations (chronic bronchitis, congenital emphysema, recurrent infections)	Risk of perioperative respiratory complications
Gastrointestinal/nutrition	
Feeding problems/failure to thrive	Risk of aspiration; possible metabolic or electrolyte disturbances
Gastroesophageal reflux	Risk of aspiration
Cardiovascular	
Congenital heart defects (ASD, VSD, PDA); heart block	Risk of hemodynamic instability or arrhythmias; adapted anesthetic management and monitoring required
Skeletal/musculoskeletal	
Skeletal anomalies (short ribs, patellar hypo-/aplasia, joint laxity/hypermobility, contractures, vertebral abnormalities such as spina bifida occulta, scoliosis, lumbar lordosis)	Positioning challenges and risk of joint injury; careful handling during positioning and transport required; implications for regional anesthesia or neuraxial techniques
Muscle weakness	Possible increased sensitivity to neuromuscular blocking agents; risk of delayed recovery; neuromuscular monitoring required
Growth/metabolic	
Growth restriction, low body weight, lipodystrophy, hypotrophy	Adjustment of anesthetic dosing and airway device size according to height/weight rather than age; careful fluid and temperature management
Neurological/developmental	
Delayed neuromotor development	Potential limited cooperation; adjustment of communication and sedation strategies accordingly
Seizures	Consider drug interactions with antiepileptics; ensure perioperative seizure prophylaxis and monitoring
Sensory	
Hearing and vision difficulties (e.g., severe deafness, myopia, strabismus)	Impaired communication and cooperation; appropriate management of hearing or visual aids during perioperative care required

We report the successful anesthetic management of a 5-year-old boy (99 cm, 12 kg) with MGS, who underwent nasopharyngeal inspection, otomicroscopy, adenoidectomy, bilateral myringotomy with insertion of tympanostomy tubes under general anesthesia in June 2025. To the best of our knowledge, this is the first published case report describing the anesthetic management of a patient with MGS.

Ethical approval for this case report (protocol number 25–174 RS) was provided by the Ethical Committee of the Department of Medicine, Philipps University of Marburg, Germany on June 11, 2025. Written informed consent to publish this case and the associated images was obtained from the parents. This report adheres to the CARE guideline for case reports [23].

## Case report

We report on a 5-year-old boy with genetically confirmed MGS, born to healthy parents. He is an only child. Pregnancy and delivery were unremarkable, with full-term birth and a birth weight of 2990 g. Clinical features associated with MGS in this patient include short stature, microtia, and developmental delay – particularly affecting speech and language development – without any observed patellar abnormalities. There is no history of cardiac or metabolic disorders. Due to chronic bronchitis, the patient intermittently uses inhaled salbutamol as needed. He is not on any other regular medications and has no known allergies. He attends a regular kindergarten and receives ongoing speech therapy.

The patient had previously undergone one general anesthetic for hearing screening, which was well tolerated and uneventful; however, no further details regarding the anesthetic procedure were available. Despite an initially unremarkable hearing screening, he continued to experience hearing difficulties that persisted despite conservative management, including decongestant nasal drops and corticosteroid-containing inhalation therapy. Otomicroscopy could not be performed due to microtia and limited patient compliance. As a result, surgical intervention under general anesthesia was now indicated.

### Anesthetic management

A pre-anesthetic examination was conducted a few days prior to the procedure. It revealed a mouth opening of approximately 2.5 cm with full neck extension, normal dentition without any loose teeth, and a Mallampati score of I. The patient underwent a routine preoperative evaluation, which did not indicate any signs of a difficult airway. He was classified as ASA physical status II. According to his parents, the child exhibited normal physical endurance. In line with institutional standards, topical lidocaine/prilocaine patches were applied preoperatively to the dorsum of both hands, and oral premedication with 6 mg of midazolam syrup was administered.

Alternative airway devices (endotracheal tubes, laryngeal masks, and oropharyngeal as well as nasopharyngeal airways) were prepared prior to arrival, with sizes selected according to the child’s weight and height, communicated within the team, and supplemented by one size above and below to ensure immediate availability. The induction was performed in the operating room with an ENT surgeon on call. All team members were briefed on the airway management plan and backup strategies prior to induction. Videolaryngoscopy was defined as the primary plan (Plan A), with a laryngeal mask airway prepared as a backup (Plan B) and a flexible bronchoscope available for fiberoptic intubation as an additional backup strategy (Plan C). Neuromuscular blockade was planned as the primary approach, supported by the immediate availability of sugammadex for rapid reversal if required.

**Fig. 1:**
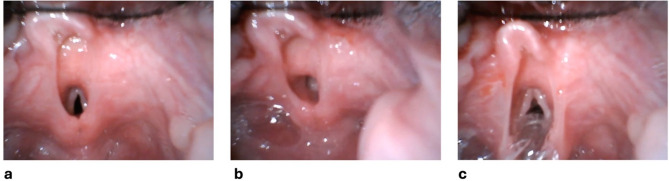
Videolaryngoscopic view of the glottis and surrounding structures during intubation.** a** The glottic opening appears anatomically deep and slightly anteriorly positioned, with mild leftward deviation of the epiglottis. Cormack-Lehane grade I view. **b** View obstructed by soft, collapsible surrounding tissue; minimal scope manipulation leads to significant visual impairment. **c** Unobstructed visualization of the vocal cords after adequate retraction of surrounding structures using a preformed 10 CH stylet to guide the endotracheal tube

The patient entered the operating room calm and well premedicated, showing no signs of anxiety. Initial vital signs were within normal limits: SpO₂ 98%, heart rate 130 bpm in sinus rhythm. A peripheral 22 gauge intravenous catheter was placed on the dorsum of the left hand. Induction of anesthesia was achieved with 80 mg of propofol 1%, 25 µg of fentanyl, and 8 mg of rocuronium. Mask ventilation was uneventful. Tracheal intubation was initially attempted using videolaryngoscopy. A Cormack-Lehane grade I view of the glottis was obtained; however, the glottic opening appeared anteriorly positioned, and the glottic plane was anatomically clearly deeper than the plane of the arytenoid cartilages, with the epiglottis slightly deviated to the left (see Fig. 1a).

To facilitate intubation, a pediatric flexible stylet was first used but proved too soft to guide the tube tip effectively through the glottis due to the altered anatomy. The laryngeal tissues appeared unusually soft and mobile; even minimal manipulation of the videolaryngoscope led to collapse of the surrounding structures, significantly impairing visualization of the deeply positioned glottis (see Fig. 1b). On the second attempt, a stiffer 10 Charrière (CH) stylet was used, and the endotracheal tube (internal diameter 4.0 mm) was pre-curved accordingly. To prevent the stylet from protruding beyond the distal tip of the tube and minimize the risk of airway injury, it was bent back and securely fixed at the tube adapter. This approach allowed successful orotracheal intubation under direct visualization (see Fig. 1c). The endotracheal tube was secured at 13 cm at the dental line. Bilateral vesicular breath sounds were confirmed by auscultation, along with positive capnography and stable vital signs.

The cuff pressure of the endotracheal tube was set to 25 cmH₂O. Anesthesia was maintained with a continuous infusion of propofol at 120 mg/h and remifentanil initially at 300 µg/h, later reduced to 150 µg/h. Depth of anesthesia was monitored using bispectral index (BIS), with values consistently ranging between 41 and 48. A pediatric electrolyte solution was administered as maintenance fluid at a rate of 10 mL/kg/h.

Pressure-controlled ventilation was uncomplicated, with a maximum inspiratory pressure (Pinsp) of 15 mbar, PEEP of 5 mbar, a tidal volume of 6–8 mL/kg, an inspiratory-to-expiratory ratio of 1:2, and FiO₂ between 0.35 and 0.5. For PONV prophylaxis, 1.8 mg of dexamethasone and 240 µg of granisetrone were administered. Postoperative analgesia was initiated with 120 mg of rectal ibuprofen following induction of anesthesia. Normothermia was preserved through active warming techniques.

Surgical time (skin-to-skin) was 25 min. At the end of the procedure, prompt recovery of neuromuscular function was observed, as confirmed by a train-of-four (TOF) ratio greater than 0.9. The patient was extubated uneventfully and transferred to the post-anesthesia care unit (PACU) with an Aldrete score of 9. He remained in the PACU for 75 min, during which he received 180 mg of metamizole for analgesia and 3 µg of dexmedetomidine as an adjunctive agent. Oxygen saturation under ambient air remained stable at 98–99%, and supplemental oxygen was not required. Upon discharge from the PACU, the patient had an Aldrete score of 14 and a pain score (NRS) of 0.

The postoperative course was uneventful, and the patient was discharged home on the same day of surgery. A routine follow-up examination with the ENT team five weeks after the procedure revealed no abnormalities.

## Discussion

This case highlights several key aspects in the anesthetic management of pediatric patients with MGS, a rare congenital disorder associated with craniofacial and skeletal abnormalities. To our knowledge, this is the first published report describing perioperative anesthetic care in a child with MGS.

Although our patient showed no predictors of a difficult airway during the pre-anesthetic evaluation and presented calm and cooperative, anxiolytic premedication was considered appropriate. Oral midazolam was administered to reduce perioperative anxiety. In general, however, midazolam may not be the ideal choice in patients with potentially difficult airways, as it can depress respiratory drive and impair airway tone – potentially increasing the risk of perioperative respiratory adverse events (PRAEs) [24].

In children with MGS – particularly when airway challenges are anticipated – alternative agents such as intranasal dexmedetomidine, alone or in combination with low-dose esketamine, may be preferable due to their favorable sedative and respiratory profiles. Intranasal dexmedetomidine is associated with a reduced incidence of PRAEs, particularly in children undergoing tonsillectomy and adenoidectomy [25]. Furthermore, compared to midazolam, dexmedetomidine has been linked to a lower incidence of emergence delirium, making it an attractive option in pediatric anesthesia [26].

Thorough facial assessment is a key component of airway management planning in patients with MGS, as characteristic craniofacial dysmorphisms – such as microstomia, retrognathic mandible, maxillary and mandibular hypoplasia, fibrous ankylosis of the temporomandibular joint, external ear anomalies, and reduced mouth opening – may be present [6, 7, 9]. More rarely, cleft palate or similar malformations can also occur (Table 1) [9, 10]. Importantly, these typical features of MGS – along with findings such as midface hypoplasia or facial asymmetry – have been associated with an increased risk of difficult face mask ventilation and laryngoscopy in pediatric patients [27].

In our patient, preoperative airway evaluation was unremarkable – with a mouth opening of 2.5 cm, Mallampati class I, and full neck extension. However, videolaryngoscopy revealed anatomical deviations characteristic of MGS, including an anteriorly positioned and deeply located glottic opening, along with slight lateral deviation of the epiglottis.

Intubation was initially attempted with a flexible pediatric stylet, which proved insufficient to navigate the glottic anatomy. On the second attempt, a firmer 10 CH stylet was used successfully. It should be emphasized that when using stiffer introducers – especially in pediatric patients – extreme caution is required to avoid injury to the delicate upper airway mucosa. Adequate lubrication, gentle manipulation, and continuous visual guidance are essential to minimize the risk of trauma, subglottic injury, or post-extubation complications.

Despite successful intubation with videolaryngoscopy in our case, alternative strategies should be considered in pediatric patients with potential airway challenges.

Fiberoptic intubation in the spontaneously breathing adult remains the gold standard for managing difficult airways; however, its applicability in children is limited by poor cooperation [27, 28]. In selected pediatric patients with an anticipated difficult airway, fiberoptic intubation through a supraglottic or nasopharyngeal airway under sedation and topic anesthesia may serve as an alternative approach [29, 30]. Although this was not required as the primary strategy in our patient – given the unremarkable preoperative airway evaluation and previously uneventful anesthetics – flexible bronchoscopy as part of a hybrid approach could have represented a reasonable secondary option, particularly after the first unsuccessful videolaryngoscopic attempt [29]. This combined technique, integrating videolaryngoscopy and flexible bronchoscopy, has demonstrated success rates comparable to other advanced airway strategies in pediatric difficult airway management [31]. Videolaryngoscopy facilitates visualization by displacing the tongue and surrounding tissues, while the flexible bronchoscope functions as a steerable stylet to guide the endotracheal tube through the vocal cords [31, 32]. The videolaryngoscopic view can also assist in overcoming resistance during tube advancement [31, 32]. However, this method requires two experienced clinicians and may not be feasible in all clinical settings [30, 32].

Besides the choice of technique, the type of videolaryngoscope blade may also influence intubation success. An age-adapted standard blade (Macintosh-type) videolaryngoscope might have been advantageous in this anatomical configuration and is often preferred in anticipated difficult pediatric airways, whereas hyperangulated blades are generally recommended as a backup in case of failed intubation with a standard blade [27, 29]. On the one hand, hyperangulated blades are commonly advocated in the presence of an anteriorly positioned larynx [29]. On the other hand, the hyperangulated blade may have hindered tube advancement due to the unusually deep anatomical position of the glottis. However, an analysis of the Pediatric Difficult Intubation (PeDI) Registry in children weighing 5 kg or more found no significant differences in the efficacy between different videolaryngoscope types [33]. In our case, we initially opted for a hyperangulated blade, as this was the videolaryngoscopy technique with which our team had the most routine clinical experience.

The use of videolaryngoscopy not only enabled precise visualization of the airway structures but also allowed external observers – such as supervising anesthesiologists, trainees, or ENT surgeons – to assess the airway in real time. This facilitated a shared understanding of the anatomical challenges and supported effective, collaborative decision-making in this complex case [27, 29, 34].

Other anesthesiologic relevant considerations in airway management of patients with MGS include, among others, the potential presence of gastroesophageal reflux (42%), congenital pulmonary emphysema (43%), bronchomalacia (33%), laryngomalacia (25%), or tracheomalacia (17%) [10], as summarized in Table 1. However, in our patient, there was no history or clinical signs of any of these conditions.

In our patient, neuromuscular blockade was achieved using rocuronium. When using non-depolarizing neuromuscular blocking agents in pediatric patients with syndromic features, intraoperative neuromuscular monitoring – as was performed in our case – is essential. Residual blockade may lead to delayed recovery and postoperative respiratory compromise, particularly in patients with craniofacial or respiratory anomalies. Objective monitoring, such as ensuring a TOF ratio >0.9 before extubation, is therefore critical to confirm complete reversal and to minimize the risk of airway obstruction or hypoventilation. Whether patients with MGS exhibit altered sensitivity to neuromuscular blocking agents remains unclear. Dose adjustment should therefore always be individualized, especially since body weight in affected individuals often falls well below age-related percentiles [11, 13].

The decision between maintaining spontaneous ventilation and using controlled ventilation with neuromuscular blockade during induction in children with a potentially difficult airway remains controversial and is influenced by multiple factors [27, 35]. While some advocate maintaining spontaneous breathing, registry data (PeDI) suggest higher rates of non-severe complications, such as hypoxemia and laryngospasm, compared to controlled ventilation with neuromuscular blockade [36]. Inadequate anesthetic depth under spontaneous breathing may further increase complication rates [36]. As an alternative, intubation with topical lidocaine and propofol-remifentanil anesthesia (without neuromuscular blockade) is sometimes proposed; however, remifentanil may precipitate bradycardia and the risk of so-called thoracic rigidity (in fact glottis closure) [27]. Ultimately, the decision must be individualized, guided by the anesthetic team’s expertise and the patient’s specific risk factors. In our patient, neuromuscular blockade was chosen as the primary strategy, since the preoperative airway evaluation was unremarkable, previous anesthetics had been described as uneventful, and sugammadex was immediately available for rapid reversal if required. Consistent with our institutional practice, patients with potentially difficult airways undergoing ENT or maxillofacial surgery are managed in the operating room with the presence or immediate availability of an ENT surgeon, ensuring rapid access to surgical airway interventions if needed.

In our patient, surgery was performed in the supine position, and musculoskeletal anomalies commonly associated with MGS – such as patellar aplasia or joint instability – were not clinically relevant. Nevertheless, joint laxity, contractures, and hypermobility or subluxation of peripheral joints require careful positioning and adequate padding to prevent perioperative injury [2, 37]. In particular, patellar hypo- or aplasia may predispose to knee joint instability and should be considered during positioning, especially in non-supine surgical settings [1].

Whether patients with MGS can be managed on an outpatient basis depends on the individual expression of the syndrome – particularly regarding airway characteristics – as well as the type and extent of the surgical procedure. In our case, extubation was uneventful, and postoperative monitoring in the PACU proceeded without complications. Given the short and straightforward nature of the procedure, interdisciplinary planning allowed for safe outpatient management.

A multidisciplinary approach is essential in the management of patients with MGS, as the phenotypic expression can vary significantly [9]. The need for preoperative consultations with other specialties should therefore be guided by the individual phenotype and the extent of clinical impairment. In cases where a difficult airway is anticipated, existing radiological findings should be carefully reviewed, and consultation with an ENT specialist should be considered on a case-by-case basis.

## Limitations

This report describes the anesthetic management of a single patient with MGS. As with all case reports, the findings cannot be generalized, and causal inferences cannot be drawn. Nevertheless, the observations may help raise awareness of potential airway challenges in similar patients and contribute to the limited literature on this rare condition.

## Conclusion

This is the first published case report describing the anesthetic management of a patient with MGS. The case highlights the anesthetic implications of MGS, underscoring the importance of early recognition of craniofacial abnormalities, comprehensive airway assessment, and intraoperative flexibility. Videolaryngoscopy proved invaluable not only for airway visualization but also for facilitating real-time team communication. In syndromic pediatric patients, individualized anesthetic strategies and interdisciplinary planning are essential to ensure perioperative safety.

## Data Availability

No datasets were generated or analysed during the current study.

## Abbreviations

ASA: American Society of Anesthesiologists
ASD: Atrial septal defect
BIS: Bispectral index
CARE (guideline): Case report guidelines
CH: Charrière
DNA: Deoxyribonucleic acid
ENT (specialist): Ear, nose and throat
MGS: Meier-gorlin syndrome
NRS: Numeric rating scale
PACU: Post anesthesia care unit
PDA: Patent ductus arteriosus
PONV: Postoperative nausea and vomiting
PRAEs: Perioperative respiratory adverse events
TOF: Train-of-four
VSD: Ventricular septal defect
